# Erythema Migrans–like Illness among Caribbean Islanders

**DOI:** 10.3201/eid1610.100587

**Published:** 2010-10

**Authors:** Anu Sharma, Sarada Jaimungal, Khamedaye Basdeo-Maharaj, A.V. Chalapathi Rao, Surujpaul Teelucksingh

**Affiliations:** Author affiliation: University of the West Indies, St. Augustine, Trinidad and Tobago

**Keywords:** Vector-borne infections, tick borne disease, erythema chronicum migrans, skin diseases, southern tick-associated rash illness, bacteria, Lyme disease, doxycycline, Caribbean, dispatch

## Abstract

Erythema migrans is the skin manifestation of Lyme disease and southern tick-associated rash illness. Neither disease is found in the Caribbean. We report 4 cases of erythema migrans of a possible emerging clinical entity, Caribbean erythma migrans–like illness.

Erythema migrans (EM), the *pathognomonic* rash for Lyme disease (LD), occurs in ≈90% of cases (*1*). During the past 30 years, an LD-like illness has been described in the southeastern and south-central United States (*2*). This illness resembles LD clinically, but patients show no evidence of infection with the etiologic agent of LD, *Borrelia burgdorferi* (*2*) or sequelae of LD (*2*). This clinical syndrome is called southern tick-associated rash illness (STARI). The vector for STARI is not found in the Caribbean. However, we report 4 cases of EM-like lesions in Caribbean Island residents.

## Case Reports

In October 2007, a 28-year-old man (student) sought care for a rash of 6 months’ duration and generalized joint pains of a few days’ duration. He had a history of major depression but was not taking medication. He reported being well until 6 months earlier, when he noticed a rash on his left leg. It began as a lesion on his thigh and spread throughout his entire left lower limb and upper limbs. He had no other symptoms.

**Figure 1:**
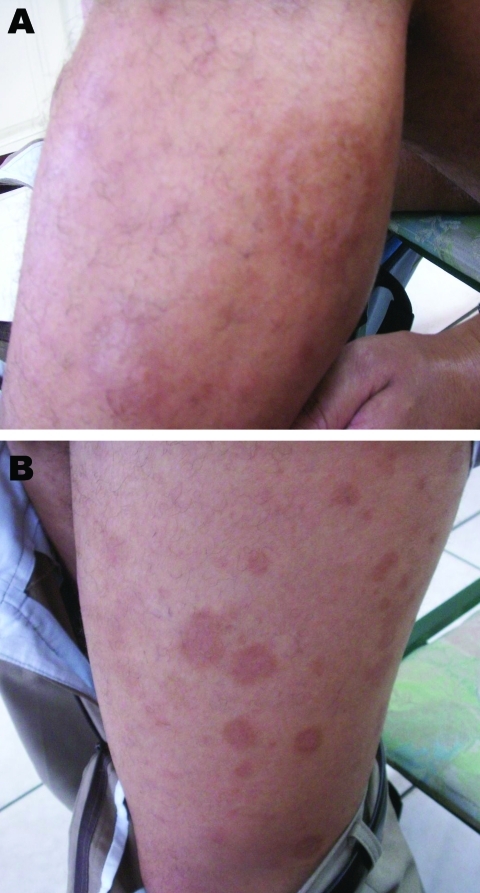
Erythema migrans–like rash on the left leg of a 28-year-old patient. A) Characteristic rash of erythema migrans (annular macular lesion that is erythematous with central clearing). B) Spread of the lesions to the rest of the leg.

The patient’s joints showed no signs of inflammation. On his left outer thigh was a large lesion (8 cm × 5 cm) with many similar lesions 1–5 cm in diameter, with central clearing on the rest of his lower limb and upper limb (Figure 1). No punctum was evident in any lesion. Two visits to a dermatologist resulted in prescriptions for topical ketoconazole and beclomethasone, which had no effect on the rash. A skin biopsy was eventually performed.

The patient grew up in St. Lucia but had traveled to Trinidad and Tobago 3 years earlier. In St. Lucia, he owned several tick-infested dogs. In 2005, he had spent 2 weeks in New York, New York, USA. He did not recall being bitten by ticks in either place.

Because of the rash and the exposure to ticks, a tick-borne illness was considered. Serologic analysis for *B. burgdorferi* and histologic analysis of a skin biopsy specimen were undertaken, and the patient was treated with doxycycline, 100 mg 2×/d for 2 weeks. Within 1 week, his arthralgia disappeared, and by week 2, the rash had resolved. At 6-month follow-up, the rash had not recurred.

**Figure 2:**
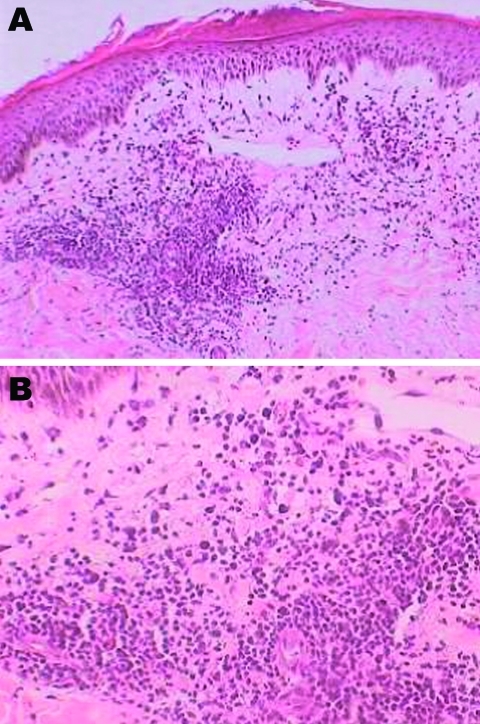
Histologic analysis of a skin biopsy specimen of a 28-year-old patient with erythema migrans, showing characteristics of erythema migrans. The epidermis shows parakeratosis, microvesicle formation, lichenoid interface, dermal edema, and perivascular chronic inflammatory cell infiltrates. Original magnification ×20 (A) and ×40 (B).

Results for immunoglobulin (Ig) M and IgG to *B. burgdorferi* by enzyme immunoassay and the C6 peptide antibody were negative. Histologic analysis of the skin biopsy specimen showed perivascular lymphocytic infiltrates with admixture of plasmocytes, consistent with EM (Figure 2). Culture and silver staining for spirochetes were not performed. On the basis of the clinical findings and negative serologic results for *B. burgdorferi*, an EM-like illness was diagnosed.

In March 2008, a 15-year-old boy from Trinidad and Tobago sought care for a red pruritic rash of 4 weeks’ duration. Atopic eczema had been diagnosed 1 year earlier. Atopic eczema was again diagnosed and topical steroid cream prescribed, but the rash persisted. On examination in April 2008, the rash was present on his arms and legs. Multiple lesions 1–6 cm in diameter all had an erythematic border with central clearing. Histologic findings on skin biopsy were consistent with EM. After treatment with doxycycline for 14 days, the rash cleared. After 1 year, it had not recurred.

In April 2009, a 60-year-old woman from Grenada with diabetes sought care for an ongoing pruritic rash of 4–6 months’ duration. The rash had an erythematous, flat border with a central clearing 7 cm in diameter but no punctum. It occurred only on her legs, first the left, then the right. Visits to several doctors resulted in prescriptions for vitamin A, vitamin E, and steroid and antifungal creams, all of which partially cleared the rash. She had no history of recent influenza-like symptoms or neurologic complaints. EM was diagnosed in October by biopsy. After treatment with doxycycline for 7 days, the rash resolved and had not recurred after 1 year.

In January 2010, a 48-year-old woman (veterinary laboratory assistant) from Trinidad and Tobago sought care for a circular, erythematic rash of 2 weeks’ duration. The rash started on her right leg; had a flat, erythematous border with central clearing; and progressively increased to 5.1 cm in diameter. There was no evidence of a punctum. Two similar 2–3-cm lesions on her left leg appeared 3 days after the first lesion. She also reported fatigue, mild body and joint pains, and headaches relieved by acetaminophen. She had no history of travel to an LD-endemic region. EM was diagnosed and treatment with doxycyline was started. The rash began clearing after 2 weeks of treatment and had not recurred after 4 months.

## Conclusions

STARI is characterized by expanding annular erythema, mild constitutional symptoms, spring–summer seasonality, recent antecedent tick bite at the site of the skin rash, absence of *B. burgdorferi* antibodies, and negative skin biopsy culture results for *B. burgdorferi* (*2*). Because none of our patients recalled an antecedent tick bite or had a punctum, their illnesses failed to meet the criteria for STARI.

In a prospective clinical evaluation of EM, lesions in STARI patients from Missouri were substantially smaller and more likely to have central clearing, and patients had a lower mean symptom score, compared with LD patients in a New York study (*3*). Although clinical findings of our patients were comparable with those of the Missouri STARI patients, our patients’ multiple, widespread, and oblong lesions with no punctum and lack of known histories of tick bite were comparable with those of New York LD patients (*3*).

Unlike LD, for which *Ixodes* spp. ticks are the vector, STARI is thought to be spread by the Lone Star tick (*Ambylomma americanum*), the most common tick parasitizing humans in the southeastern and south-central United States (*4*). It is not an effective carrier of *B. burgdorferi*; however, 2% of the species are infected with another spirochete, *B. lonestari* (*5*). In 1 isolated case, *B. lonestari* was identified by PCR as the etiologic agent of STARI (*6*). In another report, no evidence of *B. lonestari* infection was found in 30 cases studied (*7*).

EM also has been associated with other infectious agents. In an imported human African trpanosomiasis case in France, the skin lesion resembled EM (*8*). In a study in Spain (*9*), *Dermacentor marginatus* ticks infected with *Rickettsia slovaca* produced EM-like rash and lymphadenopathy in 22 patients studied. In addition, EM has been reported as a skin manifestation in Rocky Mountain spotted fever (*10*). EM can no longer be considered solely a sign of borrelial infection.

*A. americanum* ticks occur mainly in North America and certain areas of Mexico (*11*). *A. americanum* ticks and the LD vectors (*I. scapularis* and *I. pacificus* ticks) are not found in the Caribbean (*11*). The vector for the EM-like rash in our cases is therefore unlikely to be that of either STARI or LD. Other *Ambylomma* spp. ticks, however, have been found in Trinidad and Tobago (*12*). Whether these species are potential vectors for EM remains unknown; therefore, whether we are describing a new entity, Caribbean erythema migrans–like illness, remains to be determined.

We report EM-like skin lesions in 4 Caribbean nationals who did not have foreign exposure to ticks. The vectors for STARI and LD are not known to exist in the Caribbean region. Further research is warranted to isolate the responsible vector and etiologic agent.
